# Mechanism of Copper Uptake from Blood Plasma Ceruloplasmin by Mammalian Cells

**DOI:** 10.1371/journal.pone.0149516

**Published:** 2016-03-02

**Authors:** Danny Ramos, David Mar, Michael Ishida, Rebecca Vargas, Michaella Gaite, Aaron Montgomery, Maria C. Linder

**Affiliations:** Department of Chemistry and Biochemistry, California State University, Fullerton, California, United States of America; Auburn University, UNITED STATES

## Abstract

Ceruloplasmin, the main copper binding protein in blood plasma, has been of particular interest for its role in efflux of iron from cells, but has additional functions. Here we tested the hypothesis that it releases its copper for cell uptake by interacting with a cell surface reductase and transporters, producing apoceruloplasmin. Uptake and transepithelial transport of copper from ceruloplasmin was demonstrated with mammary epithelial cell monolayers (PMC42) with tight junctions grown in bicameral chambers, and purified human ^64^Cu-labeled ceruloplasmin secreted by HepG2 cells. Monolayers took up virtually all the ^64^Cu over 16h and secreted half into the apical (milk) fluid. This was partly inhibited by Ag(I). The ^64^Cu in ceruloplasmin purified from plasma of ^64^Cu-injected mice accumulated linearly in mouse embryonic fibroblasts (MEFs) over 3-6h. Rates were somewhat higher in Ctr1+/+ versus Ctr1-/- cells, and 3-fold lower at 2°C. The ceruloplasmin-derived ^64^Cu could not be removed by extensive washing or trypsin treatment, and most was recovered in the cytosol. Actual cell copper (determined by furnace atomic absorption) increased markedly upon 24h exposure to holoceruloplasmin. This was accompanied by a conversion of holo to apoceruloplasmin in the culture medium and did not occur during incubation in the absence of cells. Four different endocytosis inhibitors failed to prevent ^64^Cu uptake from ceruloplasmin. High concentrations of non-radioactive Cu(II)- or Fe(III)-NTA (substrates for cell surface reductases), or Cu(I)-NTA (to compete for transporter uptake) almost eliminated uptake of ^64^Cu from ceruloplasmin. MEFs had cell surface reductase activity and expressed Steap 2 (but not Steaps 3 and 4 or dCytB). However, six-day siRNA treatment was insufficient to reduce activity or uptake. We conclude that ceruloplasmin is a circulating copper transport protein that may interact with Steap2 on the cell surface, forming apoceruloplasmin, and Cu(I) that enters cells through CTR1 and an unknown copper uptake transporter.

## Introduction

Copper is a trace element required for a wide variety of enzymatic reactions critical to most living cells and for the functions of an ever-growing number of other proteins, especially in mammals, whose function is less understood [1–15]. Examples include cytochrome c oxidase (in electron transport) critical to aerobic respiration and oxidative phosphorylation; dopamine monooxygenase, on the pathway for production of catecholamines; peptidyl glycine alpha hydroxylating monooxygenase (PAM), which modifies neurohypophyseal peptide hormones; lysyl oxidase, necessary for maturation of extracellular collagen and elastin; tyrosinase, which catalyzes the polymerization of tyrosine metabolites to form melanin, in melanocytes; and intra and extracellular Cu/Zn superoxide dismutases (SOD1 and 3) and ceruloplasmin which help to neutralize reactive oxygen species [1,11,15,16,17]. Ceruloplasmin (Cp) the main Cu-containing blood plasma protein also has other functions. These include the ability to oxidize Fe(II) (ferroxidase activity)–implicated in the mediation of iron efflux from certain cells [18], and the oxidative inactivation of NO [12] and some biogenic amines (like catecholamines and serotonin) [1,13,19–22]. In addition, there is long-standing evidence that the copper in Cp enters tissues and cells (see later), implying it is a copper transport protein in the circulation.

This latter aspect of Cp function has not been pursued for some time, having been overshadowed by a focus on Cp as a ferroxidase [18,23]. Its role as a ferroxidase is thought to mediate efflux of iron from cells, since Fe(II) arriving on the cell surface through the transporter, ferroportin, cannot bind its plasma transport protein, transferrin, without first being oxidized. (Transferrin carries 1–2 atoms of Fe(III)). This is supported by data showing accumulation of iron in certain cells and organs in humans and animals lacking Cu-containing, enzymatically-active Cp [14,23,24], and by evidence that Cp physically interacts with transferrin [25]. While the ferroxidase function of Cp is of great interest, the proposed mechanism by which Cp supports cellular iron release is not without some concerns and apparent contradictions. Cp does play a role in the transfer of Fe(II) to blood plasma transferrin from some cells–like hepatocytes [14,15], but not others–like enterocytes, where this is mediated by the membrane-tethered homolog of Cp, hephaestin [26]. During the acute phase response of inflammation, Cp synthesis and its concentration in the blood increase [15]. However, this does not stimulate cellular iron efflux. In fact, transport of iron by transferrin is reduced [27,28]. Lack of Cp expression (as in genetic aceruloplasminemia) or activity (as in severe copper deficiency) does result in iron overload in certain tissues (like brain, liver, and pancreas) [23]. However, this accumulation takes a long time to develop (by age 45–55 in humans), and we would not expect that to be the case if Cp ferroxidation were essential for iron efflux. A great deal of iron in aged red blood cells is processed daily by macrophages in spleen and liver (and bone marrow–about 22 mg in the average adult) and this must be returned to the bone marrow for incorporation into new reticulocytes [29]. If this depended upon Cp ferroxidation, iron overloading would be rapid, which isn't the case. Possibly, other circulating ferroxidases detected in the blood plasma are taking up the slack and substituting for Cp (in the absence of active Cp) [30,31], but that would detract from viewing Cp as an essential ferroxidase.

Although Cp is mainly in blood plasma it is also present in some other body fluids (cerebrospinal, amniotic) and milk [15,32]. A single gene encodes expression of 3 forms of the protein (differing by only a few amino acids) through alternative splicing of the C-terminal exon of the transcript. The main (soluble, plasma/milk) form contains 1046 amino acids; one 4 amino acids longer is selectively expressed in placenta [33]; and there is a GPI linked version with 30 alternative amino acids substituting for the last 5 of the main plasma form, marking it for GPI linkage [34,35]. Total mass is about 120 kDa of protein with 12 kDa of N-linked carbohydrate. X-ray crystallography of the Cu-containing (holo) protein shows a compact structure (about 214 x 85 Å) with 3 sequential homologous regions that each have two parts (thus 6 domains) [19,36]. It has 6 tightly bound Cu atoms of three types that are found in other (mostly invertebrate) “blue” multicopper oxidases [13, 15, 19, 30, 36]. Three copper ions–two in standard Type 1 (blue) sites the other in a site lacking the typical methionine ligand, are associated with domains 2, 4 and 6. The three additional copper ions are in a trinuclear cluster are between domains 1 and 6, bound to amino acid residues from the N and C terminus, and exhibit properties of Type 3 and Type 2 copper sites depending on oxidation state [13,19]. Electrons from substrates or radicals are received mainly by the Type 1 copper in the blue sites (particularly in domains 4 and 6) and transferred to the trinuclear copper cluster for reduction of oxygen. (The whole process is mediated by at least 4 of the 6 copper atoms [13,15].) Sites for the binding of the various substrates that Cp oxidizes are in different portions of the molecule, confirming its enzymatic multifunctionality. *In vitro*, the copper in Cp cannot be removed without harsh treatments involving reduction, chelation and partial or full denaturation. In physiological solutions, Cp-Cu is not dialyzable or exchangeable [1,15–17,37]. This indicates that a major change in conformation must occur for the copper in Cp to be released. This is likely to require interaction with other proteins (such as those on the cell surface, as we have postulated).

Present knowledge indicates that Cp accounts for at least half the Cu in the blood plasma [38,39,40]. In contrast, albumin and transcuprein (a macroglobulin) are the main components of the exchangeable plasma Cu pool and the first to be labeled when traces of radioactive Cu(II) enter or are directly added to blood plasma [1,15–17,41]. Albumin (about 69 kDa) has a very high affinity Cu(II) site at its N-terminus (Kd about 10^−12^ M) in most mammals [42,43]; and transcuprein, which in humans is alpha-2-macroglobulin, has an even higher Cu affinity [44,45]. As shown with radiotracer, no copper ions initially associate with amino acids or other components of low molecular weight in blood plasma [16]. (As is the case in cells, Cu ions are always bound to proteins or peptides, and not “free” in cell or body fluids [46].) From radiotracer studies in rats [1,15–17,38,47] and stable isotope studies in humans [48] we know that dietary Cu entering the blood first binds to albumin and transcuprein; these readily exchange Cu with each other and rapidly deliver most of it to the liver and kidney [41,48]. Thus, tracer rapidly disappears from blood albumin and transcuprein, simultaneously accumulating mainly in liver and kidney in a precursor-product relationship. In hepatocytes, Cu enters Cp in the trans Golgi network [49], after being shuttled by the cytosolic Cu chaperone (ATOX1) to the Cu “pump” ATP7B (the “Wilson disease protein”) in TGN membranes [2,50]. Re-appearance of radiotracer in blood plasma, on Cp, coincides with its entry into most other organs [41], implying that at least normally, Cp is a major circulating Cu source for cells. However, although Cp may normally have this role, copper distribution is for the most part relatively normal when ceruloplasmin expression is abrogated, as demonstrated in humans with genetic aceruloplasminemia [14,51] and mice in which Cp expression has been knocked out [23,52]. Thus there appears to be physiological redundancy: albumin and transcuprein can substitute for the copper transport function of Cp in its absence [45], except that there appear to be consequences at least for delivery of copper to the fetus [37].

As shown by intravenous infusion of the purified ^67^Cu-labeled Cp (rather than ionic ^67^Cu(II)), ^67^Cu from Cp accumulated in most organs/tissues of virgin, pregnant and tumor bearing rats over time [53,54]. However, the I^125^-labeled protein moiety of Cp did not accumulate in tissues nearly as rapidly [53]. The same was found in studies by Percival and Harris [55,56] using heme-treated K562 cells and purified human Cp, and implies that Cp-Cu enters cells independently of Cp protein and thus not by endocytosis. Cu coming from Cp was internalized, since it passed through the placenta to the fetus [54] and was tracked into intracellular compartments in K562 cells [55,56]. Inhibition of Cp synthesis with cycloheximide (a general protein synthesis inhibitor) in whole animals markedly reduced incorporation of ^67^Cu into plasma Cp (leaving more in the liver) and markedly reduced radiotracer uptake by the placenta and fetus [54], indicating that synthesis and incorporation of ^67^Cu into Cp were involved in the ^67^Cu delivery. Studies by Harris and his group [55–57] using copper-deficient chick aorta or K562 cells, and McArdle and his group [58] using placental brush border vesicles provided additional evidence supporting the donation of Cu from Cp to cells. The results of all these studies led to the conclusion that Cp is a Cu transport protein that directly or indirectly delivers Cu to cells in various organs, and particularly to certain ones such as those of the placenta.

As concerns the mechanism involved, the concept that Cp-Cu is delivered by direct interaction of Cp with the cell plasma membrane is supported by evidence of Cp “receptors” from research by several independent investigators i.e. there is specific binding of Cp to a variety of whole cells [26,59–62] and plasma membrane fractions [58,63–65]. (In these studies, most of the binding of labeled Cp was specific, in that it could be inhibited by a large excess of unlabeled Cp.) Binding of Cp to specific sites on the plasma membrane would be expected if it directly delivered copper to a transmembrane transporter. This membrane transporter could be CTR1 (the only currently identified copper uptake transporter in mammals [1–3]. As already described, the copper in Cp per se is internal to its structure and cannot be removed by dialysis under normal conditions. Thus, one would postulate that an interaction of Cp with CTR1 or other components of the cell membrane would be necessary to unfold it to allow copper release. Also, prior evidence suggests Cp-Cu and Cp-protein are separated during copper delivery. Thus, we would expect that conversion of Cu-containing (holo) Cp to copper deplete (apo) Cp would be occurring at the outer cell surface. This would also help to explain why a substantial proportion of plasma Cp is always in the apo form [38,39,66]. The purpose of the research reported here was to test these concepts and provide unequivocal evidence that plasma Cp is or is not a copper transport protein.

## Materials and Methods

### Cell lines and culturing

Human hepatoma cells (HepG2) from ATCC and mouse embryonic fibroblasts expressing and not expressing copper transporter 1 (Ctr1) (kindly provided by Dennis Thiele, Duke University [67]) were cultured in MEM supplemented with 1 mM sodium pyruvate, 0.1mM non-essential amino acids, and 10 or 20% fetal bovine serum (FBS). PMC42 cells, a human mammary epithelial cell model [45], were cultured in RPMI-1640 and 10% FBS. For uptake studies, these were plated on Matrigel coated 12-well Transwell plates (in bicameral chambers), and used after resistance of the monolayers exceeded 300 Ohms, as previously described [45]. Fibroblasts were plated in 6-well plates. Fibroblasts were depleted of copper with 1 mM bathocuproine disulfonate (BCS), PMC42 cells with 120 μM tetraethylenetetraamine (Teta). In some cases, 70–80% confluent HepG2 cells were cultured in serum-free medium (Opti-MEM; Hyclone, Life Technologies) containing tracer ^64^Cu-nitrilotriacetate (NTA), for 1–2 days, to produce secreted ^64^Cu-ceruloplasmin.

### Ethics statement and preparation of ^64^Cu-ceruloplasmin

All animal work was conducted according to federal/National Institutes of Health guidelines. The Institutional Animal Care and Use Committee, California State University Fullerton, specifically approved the study (permit 13-R-04). All research involving human samples was in accordance with established ethical guidelines. Blood from human volunteers was obtained under a permit from the California State University Fullerton Institutional Review Board (CSUF IRB) entitled “Copper and iron transport and storage proteins in blood plasma or serum: Structure, function and regulation”. The Chair of the CSUF IRB and the Regulatory Compliance Coordinator of the CSUF IRB specifically approved this study, and the specific “Informed Consent” form. Participants gave written consent by signing that form, and the forms have been kept for the record, under lock and key.

Human ^64^Cu-Cp was purified from secretions of HepG2 cells cultured in serum-free medium with ^64^Cu-NTA, using DEAE chromatography, as previously described [40]. Mouse ^64^Cu-Cp was purified from blood plasma of mice injected i.p. 7–9 h earlier with 100–200 μCi ^64^Cu-NTA. Non-radioactive Cp was purified similarly from mouse blood plasma or that of human volunteers. ^64^Cu tracer was obtained from the Mallinkrodt Institute of Radiology at Washington University, St Louis. Mice were housed in plastic cages with more than the required minimal space per mouse; had bedding and objects in which to hide; were exposed to 12 h each of light and dark per day; and fed Purina rodent chow and water ad libitum. For preparation of ^64^Cu-labeled Cp, groups of 10 ^64^Cu-injected male and/or female mice (C57BL6) 2–6 mo of age were euthanized by exsanguination from the hepatic portal vein under pentobarbital anesthesia and injection of heparin. ^64^Cu-Cp was purified from fresh blood plasma by DEAE chromatography, starting with 100 mM K phosphate, pH 6.8, and eluting the Cp with a gradient from 100–300 mM K phosphate, pH 6.8, after thorough washing with the initial buffer [40]. Two-three fractions containing the very highest levels of ^64^Cu or Cp p-phenylenediamine (pPD) oxidase activity (determined as previously described [30]) were pooled and dialysed into HEPES buffer (130 mM NaCl, 10 mM KCl, 50mM Hepes, 5 mM glucose, 1 mM CaCl_2_ and 1 mM MgSO_4_, pH 7.4) and/or unsupplemented MEM culture medium, for use in the uptake studies. Analysis of the purified Cp by native PAGE stained showed two major bands (apo and holoCp; confirmed by Western blotting, mass mapping, and ^64^Cu radioactivity) in about equal proportions, plus a contaminant (apolipoprotein E) determined by mass mapping at the Mass Spectrometry Facility, Department of Chemistry, University of California San Diego) that accounted for about 10% of the total protein. No other known Cu-containing proteins were present, including albumin and transcuprein/α_2_-macroglobulin (as determined by size exclusion chromatography and Western blotting). Thus Cp was the only potential source of ^64^Cu for the cells. Concentrations of holoCp in the solutions were expressed in terms of ng/ml Cp-Cu (based on measurements of actual Cu using furnace atomic absorption) and/or pPD oxidase activity (pmol/min/ml).

### Cu uptake studies

Rates of Cu uptake from Cp were determined by exposing the basolateral (“blood”) side of PMC monolayers to ^64^Cu-Cp and determining uptake of ^64^Cu into cells as well as its further transfer into the apical medium over 1–24 h, as described previously for other copper sources [45]. In the case of the PMC42 cell monolayers, ^64^Cu-Cp was provided to the basolateral chamber in HEPES buffer (see previous paragraph). In the case of mouse embryonic fibroblasts (MEF), uptake rate was measured as the linear accumulation of radioactivity in cells alone (in 6 well plates) over 3 h or more in unsupplemented MEM containing the purified ^64^Cu-Cp. The specific activity of the purified ^64^Cu-Cp to which cells were exposed varied from experiment to experiment, depending upon the amount of Cp purified, the number of cell samples to which it was exposed, the dose of radioactive copper injected into the mice, and the state of decay of the ^64^Cu (half-life 12.8 h). Cp-Cu concentrations (measured by furnace atomic absorption) were much less variable and usually in the range of 0.3 μM; Cp concentrations measured as enzyme activity (with p-phenylenediamine) were usually about 4 pmol/min/ml. Since holoCp (the form donating copper) contains 6 copper atoms/molecule, Cp protein concentrations were about 1.8 μM (6 x 0.3 μM Cp-Cu). Concentrations of radioactive copper (in Cp) to which cells were exposed ranged from about 10,000 to 200,000 cpm/ml. Thus, the specific activity of the ^64^Cu-Cp ranged from ~5–110 x 10^12^ cpm/mol. In most uptake studies, cells in 6 well plates were incubated with ~0.6 nmol of holoCp/well in 2 ml of medium, and 1–2% of the copper was absorbed over 3 h. Radioactivity was counted in a Perkin Elmer model Cobra II gamma counter that automatically corrected for ^64^Cu half-life. Samples were counted at least twice for 1 min (or 5 min when counts were below 1000), averaged, and blanks (usually zero) were subtracted. Usually, rates of uptake were corrected for cell number based on protein analysis of lysates by the BCA assay (Thermo Scientific Pierce), using bovine serum albumin as the standard, and are expressed as percent dose/h/mg cell protein, or variations thereof, so that data from two or more studies (with variations in Cp-Cu, ^64^Cu-Cp and Cp pPD oxidase specific activities) could be combined. In both systems, cells or monolayers were washed 3X with warm MEM prior to initiation of uptake studies, and 3-8X with ice-cold MEM, phosphate buffered saline (PBS) pH 7, and/or isotonic saline buffered with Na acetate, pH 5.0 or 3.0, prior to cell collection. In some cases, cells were incubated for 5 and 60 min with trypsin (3%, which is 3X the normal concentration used for cell passage) prior to washing with 1% BSA in 0.9% NaCl. Usually, cells were collected by dissolving in lysis buffer (150 mM NaCl, 20 mM Tris, and 1% Triton X-100, pH 7.5), as previously described [45]. To determine whether ^64^Cu from Cp was in the cell cytosol, scraped/pipetted/dispersed cells in MEM were broken open by nitrogen bomb cavitation or by mixing with 1:5 diluted (hypotonic) PBS, and the cytosol and pellet were separated after ultracentrifugation for 1h at 105,000 x g (4° C). In some cases, non-radioactive 150 μM Cu(II), Cu(I), or Fe(III) as the NTA complex (1:5 molar ratio), was added during the uptake phase, or Ag(I) (5 or 50 μM AgNO_3_) was added to inhibit uptake of Cu by CTR1 [68]. Effects of endocytosis inhibitors were examined by measuring rates of Cu uptake from Cp over 3 h (after 1 h preincubation) in the absence and presence of fluorosulfonyl benzoyladenosine (FSBA; 100 μM), nocodazole (5 μM), Pitstop 2 (12 μM; Abcam), and/or DynaSore© 80 μM; Sigma Aldrich). Control cells were given equal volumes of the vehicle (DMSO) in which the inhibitors were dissolved, 100 and 0.45 μl per ml, for FSBA and nocodazole, respectively. With Pitstop 2 and DynaSore, 21 and 100 μl of 2% DMSO were added per ml of medium, respectively.

### Cu analysis

This was by graphite furnace atomic absorption spectrometry using a Perkin Elmer 4110 ZL Zeeman instrument. Samples of cell extracts were wet-ashed in trace element grade nitric acid and the mineral residue dissolved in 1% nitric acid prior to Cu analysis.

### Detection of apo and holo Cp

Small portions of Cp used for uptake studies after purification by DEAE chromatography (see *Preparation of *^*64*^*Cu-ceruloplasmin*) were separated in 4% native (slab) PAGE gels, transferred to PVDF membranes, blocked with 5% milk proteins, and probed with primary antibody against human Cp (goat anti human Cp; Sigma), and secondary antibody (rabbit anti goat IgG) conjugated with ALP (Sigma). Western blots were developed with alkaline phosphatase developing buffer and substrates (500 μl NBT, 500 μl BCIP added to 50 ml of 100 mM TrisHCl, pH 9.4 containing 1.5 mM MgCl_2_), imaged with the Enduro GDS imaging system, with analysis of the images by ImageJ processing and Java analysis software.

### Quantitation of mRNA and RNAi treatments

Quantitative Real Time PCR was performed on cDNA samples prepared from total RNA extracted from cells by the RNA-Bee RNA isolation reagent (Tel-Test; Friendswood, TX), using the BioRad iCycler and TaqMan technology with primers and probes. Primers and probes for murine dCytb and Steaps 2, 3 and 4, were designed and provided by Applied Biosystems (Carlsbad, CA): TaqMan Gene expression assay Nos. Mm01335931_m1 (dCytb), Mm00459312_m1 (Steap 2), Mm0287243_m1 (Steap 3), and Mm00475405_m1 (Steap 4), respectively), to be compared with 18S rRNA (Mm03928990_g1). siRNA targeting murine Steap 2, and scrambled (scr) siRNA were designed and provided by Dharmacon (LaFayette, CO) (L-054348-00-0005). siRNA or scrRNA (1 μl 25 nM, in 3 μl Dharmafect; Dharmacon) was delivered to wells each seeded with 100,000 or 200,000 cells that had been growing for 2 days. In some cases, additional si/scrRNA treatments were given 3 days later (in new medium). Expression of mRNA was calculated by comparing cycling time (Ct) values relative to those of 18 S rRNA in the same sample as follows: Ct for reductase mRNA minus Ct for 18 S rRNA = ∆Ct; target mRNA expression = 2^^(-∆Ct)^ [2^(-∆Ct)].

### Measurement of cupric and ferric reductase activity on the cell surface

The assay developed by Wyman et al [69] was applied, as previously described [70], by measuring the formation of Cu(I) and Fe(II) from 50 μM Cu(II)- or Fe(III)-NTA over 1–3 h by whole cells at 37°C, in the presence of 200 μM bathocuproine disulfonate (BCS) or ferrozine, respectively.

### Statistics

Data are reported as Means ± SD (N). Statistical significance for comparison of two kinds of samples was determined by unpaired two-tailed student’s t-test, that for differences in slopes (slope vs no slope) by regression analysis coupled with residual diagnostics that included residuals plots to test for constant mean and constant variance; normal QQ plots as well as the Shapiro-Wilk test to investigate their normality; and residuals plots along with sample autocorrelation function calculation, to check uncorrelatedness. p values <0.05 were considered significant.

## Results

### Uptake and overall transport of human Cp-Cu by human mammary epithelial cell monolayers

**Fig 1:**
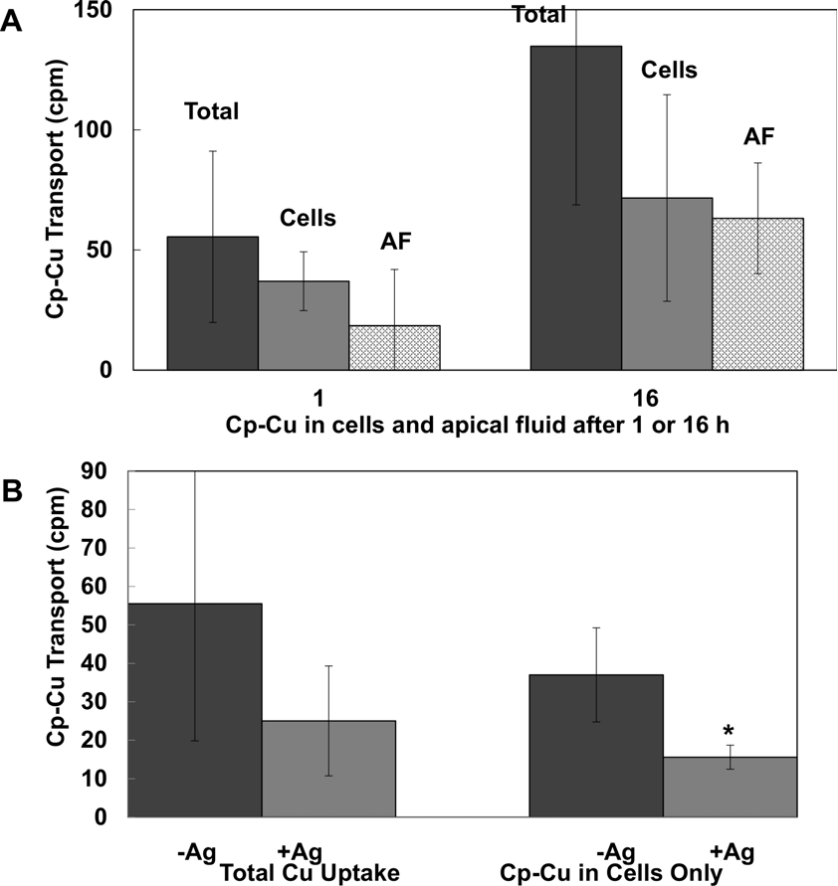
Uptake and transepithelial transport of ^64^Cu from human ceruloplasmin (Cp) by monolayers of a human mammary epithelial cells (PMC42). PMC42 monolayers with tight junctions (resistance > 300 Ohms), grown in bicameral chambers, were exposed to ^64^Cu-Cp purified from secretions of HepG2 cells treated with ^64^Cu-NTA, on the basolateral (“blood”) side, and radioactivity (cpm) in washed cells (“Cells”) and apical fluid (“AF”) was measured. Total Cu uptake (“Total”) was the sum of radioactivity in cells plus AF. (A) Total uptake, and radioactivity in cells and AF after 1 and 16 h of incubation. (B) Total uptake and cell accumulation of ^64^Cu from Cp after 1 h, in the absence and presence of Ag(I) (50 μM). Data are Means ± SD, N = 4. *p<0.01 for Ag effect.

As a first attempt to demonstrate that cultured cells could take up copper from plasma Cp, we produced ^64^Cu-labeled human Cp by exposing human hepatoma cells (HepG2) to ^64^Cu-NTA, collecting secretions, and purifying the Cp by anion exchange chromatography. Mammary epithelial cell monolayers (PMC42) with tight junctions, grown in Transwell plates, were then exposed to the purified ^64^Cu-Cp on the “blood” side of the monolayer, and the radioactive Cu taken up by the cells and transferred to the apical fluid (on the “milk” side of the monolayer) was monitored. The results of two such experiments for cells exposed for 1 and 16 h are given in Fig 1A. In both cases ^64^Cu from Cp was taken up by the cells, and some was released on the other side of the monolayer. Total uptake (radioactivity in cells and apical fluid), radioactivity in the cells, and the proportion in apical secretions all increased with exposure time, as would be expected for an uptake process (as opposed to simple adherence to the outer side of the plasma membrane). It is noteworthy that by 16 h, all of the radioactive copper in ^64^Cu-Cp had been removed from the basolateral fluid, indicating that all the copper in Cp had been taken up. It is also noteworthy that the radioactivity of the labeled Cp produced by the HepG2 cells used on and recovered from the PMC42 cell monolayers and apical fluid was not very high; and ^64^Cu has a very short half-life (12.8 h). Thus, the ^64^Cu cpm recovered in cells and fluids was low (about 1000 cpm), and this contributed to variability. However, radioactivity was clearly recovered in the cells and apical fluid, indicating uptake and transepithelial transport of Cu coming from Cp.

To indicate whether CTR1 was involved, uptake of copper from HepG2-cell-produced ^64^Cu-Cp by PMC42 cells was determined in the presence and absence of 50 μM Ag(I), which inhibits this transporter [45,68]. Though variable, the results showed some inhibition by silver ions (Fig 1B), suggesting that some but not all uptake of Cu by these cells was mediated by CTR1 (or another Ag-inhibitable transporter). Again, ^64^Cu cpm were very low and variable, but clearly present in cells and apical fluid.

### Uptake of mouse Cp Cu by mouse embryonic fibroblasts expressing and not expressing Ctr1

Since it was difficult to get sufficient Cu-radiolabeled Cp from the HepG2 cell secretions, and since it is virtually impossible to radiolabel purified ceruloplasmin *in vitro*, we decided to use rodents to produce the ^64^Cu-labeled protein, and use it on cell lines. To simultaneously address the question of whether there might be a specific relationship between CTR1 and ceruloplasmin, we obtained mouse embryonic fibroblasts (MEFs) that did and did not express Ctr1 (kindly provided by Dennis Thiele, Duke University), and used purified plasma Cp from ^64^Cu-injected mice, which provided larger amounts of Cp and greater labeling (cpm). (Cp was 90% pure (see Methods), and Cp was the only component labeled with ^64^Cu, as determined by size exclusion chromatography, native PAGE and autoradiography.) Concentrations of radiolabeled holoCp to which cells were exposed were generally about 10-fold lower than those in normal mouse plasma (see later), and in the range of 0.3 μM Cp-Cu (based on analysis by furnace atomic absorption) or 4 pmol/min/ml pPD oxidase activity (see Methods).

**Fig 2:**
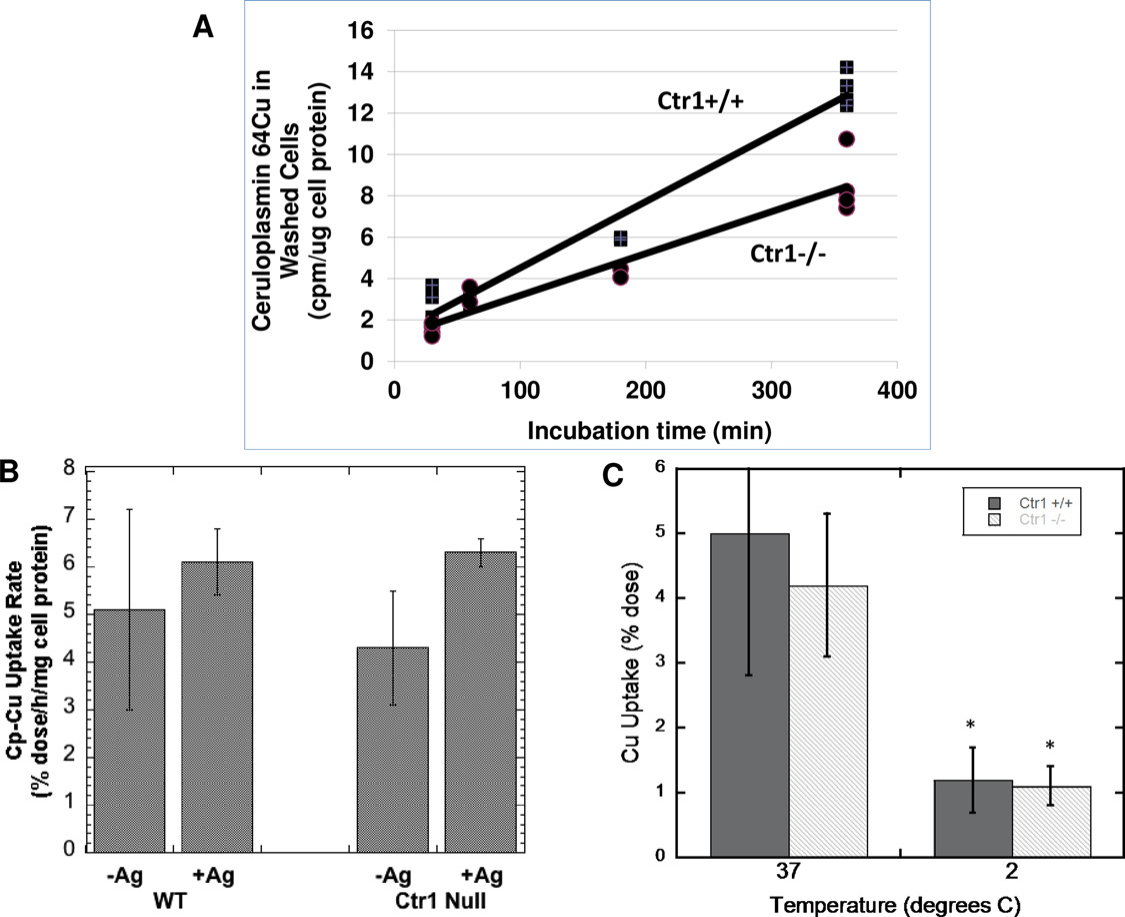
Uptake of ^64^Cu from mouse Cp by mouse embryonic fibroblasts (MEF) expressing (Ctr1+/+; WT) and not expressing (Ctr1-/-; Null) Ctr1, and effects of Ag(I) or cooling to 2° C. (A) Time course of uptake over 6 h, measured in terms of cpm per μg cell protein, showing results for cells in individual 6 well plates (representative data for multiple studies). The difference in slope of the lines for the WT and null cells was statistically significant (p<0.001) by model-validated regression analysis (see Methods). (B) Effect of Ag(I) (5 μM) on the uptake rate of Cu from Cp over 3 h (%dose/h/mg cell protein). Means ± SD (N = 3). [A lower concentration of Ag(I) was used than for the PMC42 cells (Fig 1B) because the MEFs lifted off the plate at higher concentration.] (C) Effect of cooling cells to 2° C during measurements of uptake over 1 h, Means ± SD (N = 6–9). *p <0.001 for difference from 37° C for both cell types.

Fig 2A shows representative results for the time course of accumulation of ^64^Cu from mouse Cp in Ctr1+/+ and Ctr1-/- MEFs. Accumulation of radiocopper appeared to be fairly linear over the 6 h examined. This is consistent with an uptake process rather than binding to the cell surface, especially since we had shown earlier with isolated plasma membrane fractions, that binding of radiocopper-labeled Cp reached equilibrium within 1 h at 4°C [64]. In these (early) MEF studies, rates of Cu uptake from Cp by the Ctr1+/+ cells were about 50% greater than for those without Ctr1. Regression analysis of the data in Fig 2A proved that the difference in slopes of the two lines (representing accumulation of Cp-Cu in the WT and null cells over time) was highly significant. Pooled data from several other studies over 3 h showed that uptake rates by Ctr1-/- cells were 63 ± 16 percent those of the wild type (100 ± 7 percent; N = 9; p<0.001). This proves that Ctr1 can contribute to copper uptake from Cp when it is expressed, and these results are consistent with earlier studies we carried out with the same cells, in which copper was provided bound to albumin and transcuprein/alpha-2-macroglobulin [70]. However, the data also prove that another as yet unidentified copper uptake transporter [67,70] is the main one involved.

Many months later, when we continued our studies on uptake of Cu from Cp however, the contribution of Ctr1 to uptake had diminished, with little or no difference in uptake rates between the WT and Ctr1-/- cells. (We attribute this to subtle changes in cell function associated with continued passaging of the same cultures over many generations, and possibly also to changes in the composition of the cell culture medium–such as the hormonal and trace element content of the fetal bovine serum.) In these later-passage cells, we found that the addition of Ag(I) (which inhibits Ctr1) also failed to inhibit uptake of Cp-Cu by the WT cells–and those without Ctr1 (Fig 2B). (Ag(I) does not bind to Cp–at least under *in vivo* conditions, although it does get incorporated into Cp over time [71].) Consistent with an uptake process, we found that incubating at 2° rather than 37° C reduced uptake about 3-fold over 3 h (Fig 2C), and this was also the case over the first hour (data not shown). (As already noted, we had previously reported that maximal binding of Cp to tissue plasma membranes at 4° C occurred within 1 h [64].)

**Fig 3:**
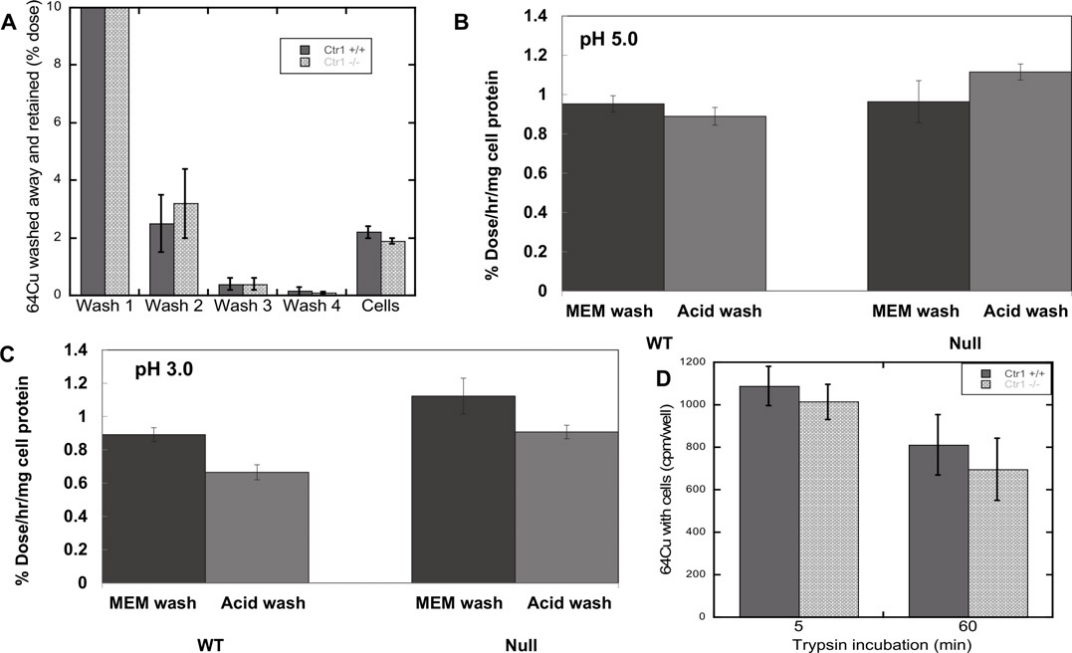
Effects of extensive washing at different pHs, and of trypsin, on retention of ^64^Cu from mouse Cp by mouse embryonic fibroblasts expressing (Ctr1+/+, WT; dark bars) and not expressing (Ctr1-/-, Null; light bars) Ctr1. (A) Combined data from several studies, showing radioactivity in washes and cells 3 h after ^64^Cu-Cp exposure, after washing extensively with phosphate buffered saline or MEM (without FBS), pH 7.0. The first wash contained 92% of the initial dose. Additional washes (beyond 4) made no difference. (B and C) Effects of washing cells 3X with MEM and then with buffers of lower pH (vs MEM): acetate buffered saline, pH 5.0 (B) or pH 3.0 (C). (D) Effects of incubating with trypsin (3%, concentrations– 3X higher than for cell culturing) over 5 or 60 min, on retention of ^64^Cu radioactivity by fibroblasts previously exposed to ^64^Cu-Cp for 3 h. Data are Means ± SD for N = 3–6 (A), N = 3 (B-D).

To further validate that we were really observing uptake and internalization of Cu from Cp rather than accumulation of ^64^Cu-Cp on the cell surface, we carried out a number of additional studies with the mouse Cp/fibroblast system, using cells incubated with ^64^Cu-Cp for 3 h. First we examined the effects of extensive washing (3-8X) with ice-cold MEM or phosphate buffered saline (PBS), pH 7. In all cases, radioactivity associated with the cells persisted, as shown by representative pooled data for 4 washes (Fig 3A). Lowering the pH of the washes to 5.0 made no difference (Fig 3B), and lowering it to pH 3.0 only reduced it about 20% (Fig 3C). Thus at most, 20% of the radioactivity was bound to the cell surface. Incubating cells with trypsin for 60 min prior to washing and harvesting also only reduced the radiocopper associated with the cells by about 25% (Fig 3D), e.g. most was within the cells.

**Fig 4:**
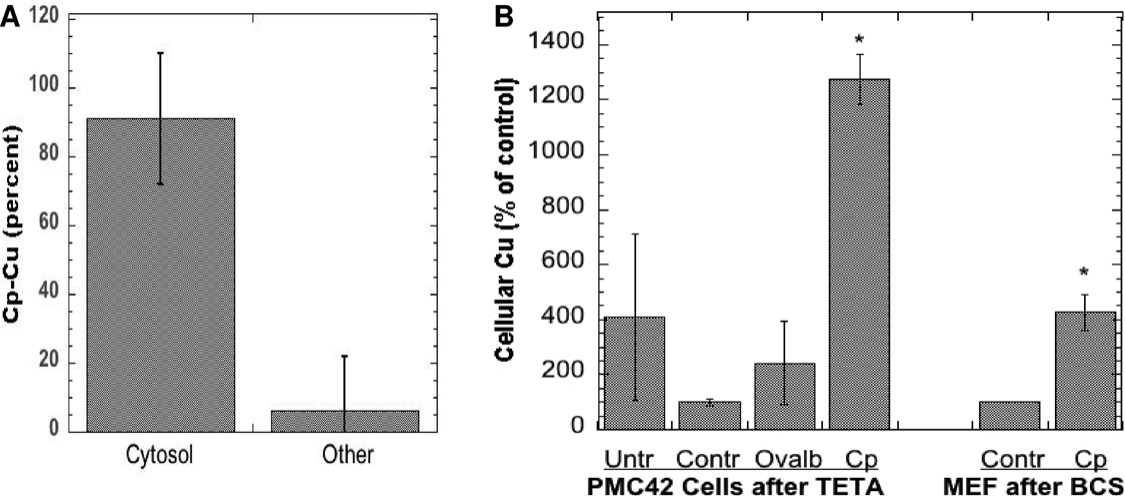
Internalization of Cp copper upon incubation with WT and Ctr1 null fibroblasts with Cp. (A) Cells were exposed to purified mouse ^64^Cu-labeled Cp for 3 h, and radioactivity (% dose) in the cell cytosol and pellet of washed cells was measured after disruption and centrifugation for 1 h at 105,000 x g. Combined data (Means ± SD; N = 8) from four experiments for both kinds of cells; cell disruption for three experiments was by nitrogen bomb cavitation, one was by exposure to hypotonic buffer. (B) Actual (non-radioactive) copper accumulated by human mammary epithelial cells (PMC42) or mouse embryonic fibroblasts (MEF; Ctr1+/+), after 6 or 24 h incubation with purified human or mouse Cp, respectively. Prior to Cp exposure, cells were pretreated for 48 h with Teta (120 μM) or BCS (1 mM), respectively, to induce copper deficiency. Controls (**Contr**) were cells treated only with chelator; **Cp** were cells treated with Cp-Cu after pretreatment with chelator; **Untr** refers to cells not treated with chelator but kept in normal MEM medium (without FBS); **Ovalb** refers to chelated cells treated with 1 mg/ml ovalbumin as another kind of control. Data are cellular Cu concentrations per mg cell protein, calculated as percent of controls in the same experiment, presented as Means ± SD (N = 3–5) for the PMC42 cells, and Means ± AD for the MEF (results of two separate individual experiments). *p <0.001 for difference from controls.

Additional evidence of internalization of the copper from Cp was obtained by looking for radioactivity in the cell cytosol, and by determining actual copper levels in the cells exposed to Cp. For the former, washed cells were disrupted and the membrane/organelle fraction was separated from the cytosol by sedimentation (centrifugation for 60 min at 105,000 x g). Cells were lysed either by bomb cavitation or by exposure to hypotonic saline. The results of 4 such studies show that almost all the radioactivity derived from incubation with ^64^Cu-Cp was in the cell cytosol and very little with the membrane fraction (Fig 4A), although some of the Cu ions may have redistributed *in vitro*, after cell disruption. Fig 4B shows that incubating cells with Cp also increased their actual copper content (determined by atomic absorption). In these studies, cells were first depleted of copper with chelating agents for 48 h (as used previously by us and others; [72,73]) and then exposed to normal medium without FBS (Controls) or that containing purified non-radioactive Cp for 6 or 24 h. The responses of two kinds of cells were examined: the mammary epithelial PMC42 cells (incubated with Cp purified from fresh human plasma), and the MEF cells expressing Ctr1 (exposed to freshly purified mouse Cp). The cells exposed to Cp clearly accumulated much more copper than those not exposed to Cp, or those exposed to other proteins or potential copper sources.

### Mechanism and kinetics of copper uptake from Cp

**Fig 5:**
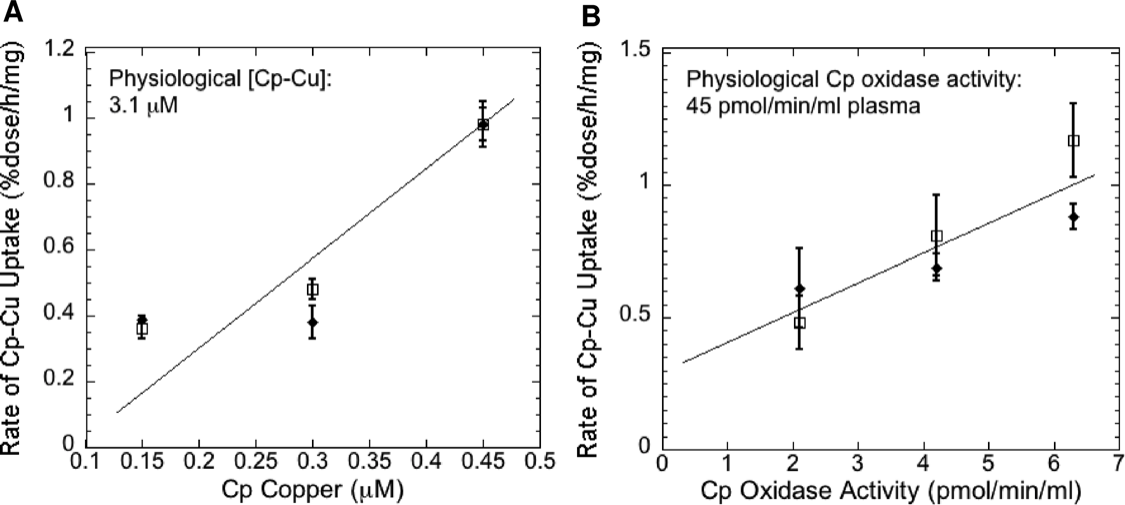
Rates of Cu uptake from Cp as a function of Cp-Cu concentrations. Purified mouse ^64^Cu-Cp samples, used to measure rates of ^64^Cu uptake from Cp by mouse embryonic fibroblasts (expressing and not expressing Ctr1), were assayed for Cu (Cp Copper; μM) and/or for pPD oxidase activity (Cp Oxidase Activity; nmol/min/mg cell protein) to provide measures of the amounts of holo Cp present. Uptake rates (%dose/h/mg cell protein) on the y-axis were plotted against Cp Copper concentrations (A) or Cp Oxidase Activity (B) on the x-axes. Independent data for Ctr1+/+ and Ctr1-/- cells are shown, points and error bars indicating Means ± SD (N = 3). Normal physiological (plasma) concentrations of Cp copper and oxidase activity in mice are indicated in the upper left corner of the graphs to compare with amounts used in the uptake assays. [Note that Cp-Cu concentrations are 2-3-fold higher in humans than mice.] For the WT (open squares) in both A and B, concentrations between the central values differed significantly from those that were higher or lower by two-tailed t-test (p<0.05–0.01). For the Ctr1-null cells, rates of uptake at the highest Cp concentrations were significantly greater than those at the lower concentrations (p<0.01). By regression analysis (with model validation), the slopes of the lines in both (A) and (B) were significantly positive (p <0.01) i.e. increased Cp correlated with increased rates of uptake.

In our studies with mouse Cp and embryonic fibroblasts, we used whatever Cp we could purify (from groups of 10 mice at a time) on a limited number of cells in 6-well plates. To determine how far away from physiological levels of plasma Cp were the Cp concentrations we were using and to begin to obtain kinetic data, we measured rates of ^64^Cu uptake from Cp over a 3-fold range of Cp concentrations, using pPD oxidase activity and Cu content of the purified Cp as a measure of Cp-Cu amount. Fig 5 shows the results of several studies using either Cp-Cu concentration determined by atomic absorption (A) or Cp oxidase activity (B) as a measure of Cp-Cu concentration (on the x-axis), relative to rate of uptake of ^64^Cu from Cp (y-axis). In both cases, rates of uptake increased in fairly linear fashion over the range of Cp concentrations employed (statistically significant positive slopes by regression analysis; p<0.01) but with no sign of even reaching half maximal rates, indicating we were far from reaching concentrations near the Vmax. Concentrations of Cp measured either way were about 10-fold lower than those in normal mouse plasma (Fig 5 “Physiological [Cp-Cu]”, “Physiological Cp oxidase activity”). These results indicate that rates of Cu uptake from plasma Cp are likely to be much higher *in vivo*.

**Fig 6:**
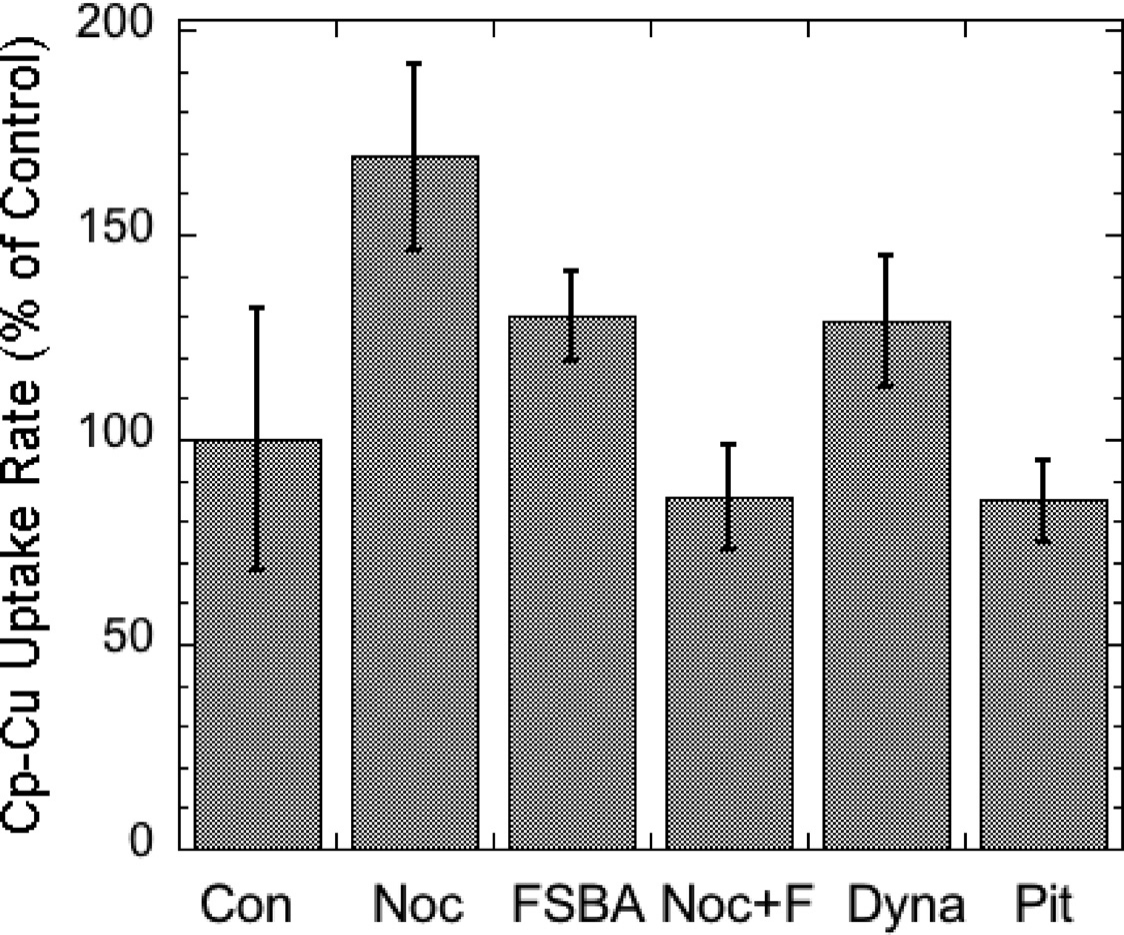
No inhibition of Cu uptake from Cp by inhibitors of endocytosis at standard concentrations: nocodazole (Noc or N; 10 μM), FSBA (F; 100 μM), Pitstop (Pit; 12 μM), or DynaSore© (80 μM). Cells were preincubated with the inhibitors for 1 h before adding the ^64^Cu-Cp. Values are uptake rates as percent of those for controls (Con) in the same experiment, Means ± SD (N = 9–7 for Con and Noc; 3–5 for the other treatments. Actual uptake rates of control (untreated cells) ranged from 1 to 2.5 percent dose/h/mg cell protein, dose being the ^64^Cu in Cp. Only Noc alone had a statistically significant effect compared to the control, and it was stimulatory.

**Fig 7:**
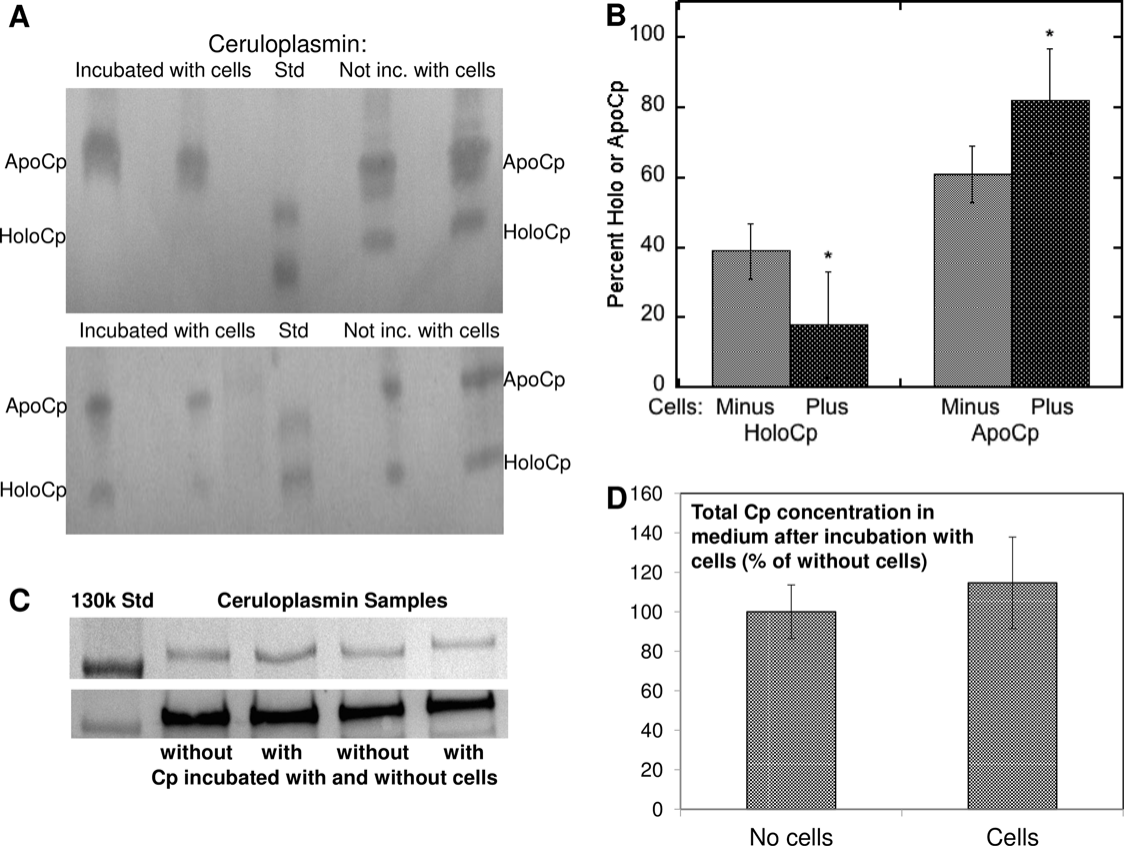
Conversion of holo Cp to apo Cp during uptake of Cu from Cp by MEF cells. (A) and (B) Purified non-radioactive mouse Cp was incubated in MEM for 24 h, without or with MEF cells expressing Ctr1, following which varying volumes of the Cp in the medium were analyzed for the proportion of apo to holo Cp by native PAGE Western blotting. High range prestained BioRad electrophoresis standards (Std) were included to identify the HoloCp and ApoCp bands. The “115” and “78” kDa standards are visible on the blots. (ApoCp migrates considerably slower than the 115 k Std; HoloCp migrates at or above the 78 k Std.) (A) Examples of Westerns. (B) Densitometric data (Means ± SD, N = 5 and 8) for blots from 4 studies, showing decrease in holo Cp and increase in apo Cp when incubated with versus without cells, *p <0.01 for difference. (C) Example of stained SDS-PAGE gel (above) and Western (below), and (D) summary of these and other densitometric data (SDS-PAGE Westerns that measure total Cp protein—both apo and holo) to determine whether exposure to cells changed/lowered total Cp concentrations over 24 h. Data are Means ± SD, N = 6. There was no statistically significant difference.

The possibility that endocytosis of Cp was involved in uptake of its copper was also examined. Treatment with four inhibitors that target different steps in the endocytosis process (Fig 6) showed that none had a statistically significant inhibitory effect on uptake of copper from Cp, indicating that (as we expected) endocytosis of Cp was clearly not involved. So we predicted that during uptake of copper from Cp by the cells, the proportion/amount of Cu in holo Cp in the medium would decline, and we found this to be the case. The proportions of holo to apo Cp in the purified Cp used for uptake studies (in MEM) was compared after 24 h of incubation in the absence and presence of MEF cells expressing Ctr1, using native PAGE Western blotting. An example of the results is shown in Fig 7A, along with a summary of the densitometric results for several blots from 4 separate studies (Fig 7B). Apo and holoCp migrate differently in native PAGE, the apo-protein being less compact and migrating more slowly than the 115 kDa BioRad SDS-PAGE standard marker, holoCp being compact and migrating faster than the same marker [38,39,40]. Fig 7B shows that when incubating the purified Cp without cells for 24 h in non FBS containing medium, about 40% was in the holo form (lighter bars); however, when incubated in the presence of cells under the same conditions, there was a large and significant decrease in the proportion of holo Cp (averaging 50%), and a concomitant increase in the proportion of apo Cp–from 60% to more than 80%. In fact, in one experiment, holo Cp completely disappeared (Fig 7A, top). (Variations in the results are to be expected, as different concentrations of the purified Cp were utilized in each case–depending on the yield of Cp and numbers of cells treated.) Incubating Cp without cells in the medium (MEM) alone did not change the proportions of apo to holo Cp detected, indicating stability of holo Cp, and so those data were combined (Control). In the Westerns, the secondary antibody (alone) did not contribute density to the regions occupied by either apo or holo Cp (data not shown). Thus during incubation with the cells, holo Cp was disappearing, as we had predicted would be the case. To determine whether this resulted in formation of additional apo Cp, we measured the amounts of total Cp (apo plus holo) in samples that were and were not exposed to the fibroblasts, using SDS-PAGE Western blotting. No significant change occurred (Fig 7C and 7D) i.e. no difference in Cp protein concentration was detected. This implies that the apo protein was being created as copper was removed from the holo form and thus, that holo Cp was being converted into apo Cp.

**Fig 8:**
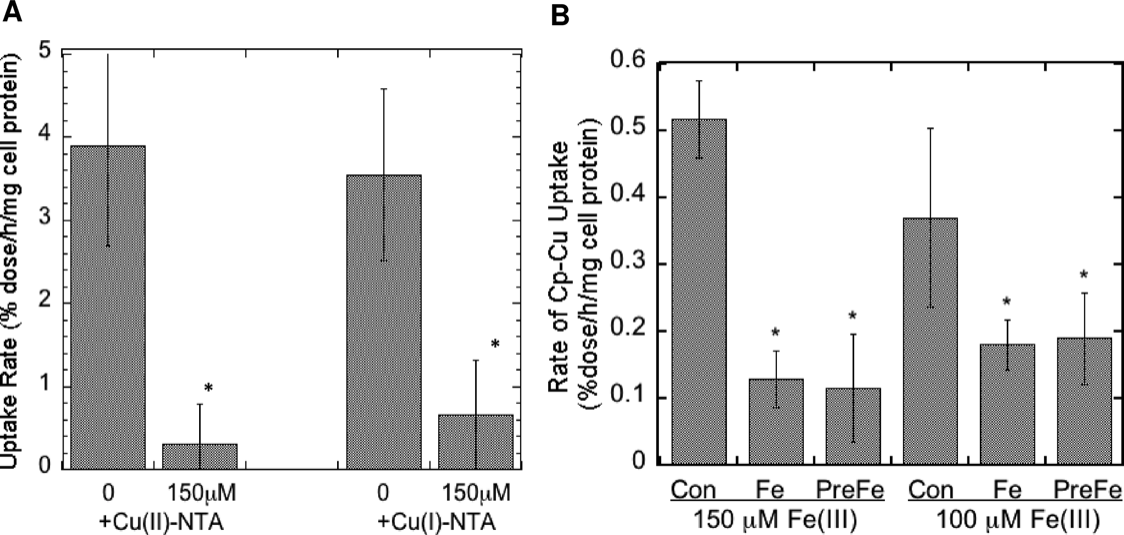
Inhibition of uptake of ^64^Cu from Cp by an excess of non-radioactive ionic Cu(II), Cu(I), or Fe(III). Uptake of ^64^Cu from purified mouse Cp over 3 h by MEF cells expressing and not expressing Ctr1 was measured in the absence and presence of non-radioactive 150 μM Cu(II) and Cu(I) (A) or Fe(III) (B) as the 5:1 NTA:metal complex. Results for cells with and without Ctr1 were similar and so were pooled, and are given as Means ± SD, N = 12 for Cu(II), 6 for Cu(I), and 6 for Fe(III). *p <0.001 for difference from controls.

The only known copper uptake transporter (CTR1) as well as the unknown uptake transporter expressed in the mouse embryonic fibroblasts we were studying [70] take up the reduced form of copper (Cu(I). However, the copper in Cp is more likely to be Cu(II) at any given moment,. Therefore we postulated that uptake of copper from Cp would need the intervention of cell surface reductases that we had demonstrated are expressed in these cells [70]. If so, an excess of non-radioactive Cu(II) would compete for reduction with the ^64^Cu(II) of mouse Cp at the cell surface, reducing cellular uptake of radiocopper from Cp. Fig 8A shows that this was the case, for both the Ctr-/- and Ctr1+/+ cells (the results of which being similar, were combined). A large excess of Cu(II)-NTA reduced uptake of radiocopper coming from Cp more than 90%, implying that reduction was a step in the uptake mechanism. Similarly, we reasoned that since the uptake transporters recognize Cu(I), an excess of non-radioactive Cu(I) would inhibit uptake of ^64^Cu(I) coming from Cp (after reduction). Again, a marked inhibition of ^64^Cu uptake from Cp by an excess of Cu(I)-NTA was observed (Fig 8A), implying competition for Ctr1 and the unknown transporter.

All the known cell surface reductases currently identified (dCytB, Steap 2, 3 and 4) also reduce Fe(III) [74]. If so, an excess of Fe(III) would most likely also inhibit uptake of copper from Cp. Fig 8B shows that this was the case. The presence of 150 or 100 μM Fe(III)-NTA (given at the same time or just before the ^64^Cu-Cp) markedly inhibited uptake of the radiocopper derived from Cp, supporting the concept that the ferric/cupric reductase is involved.

**Fig 9:**
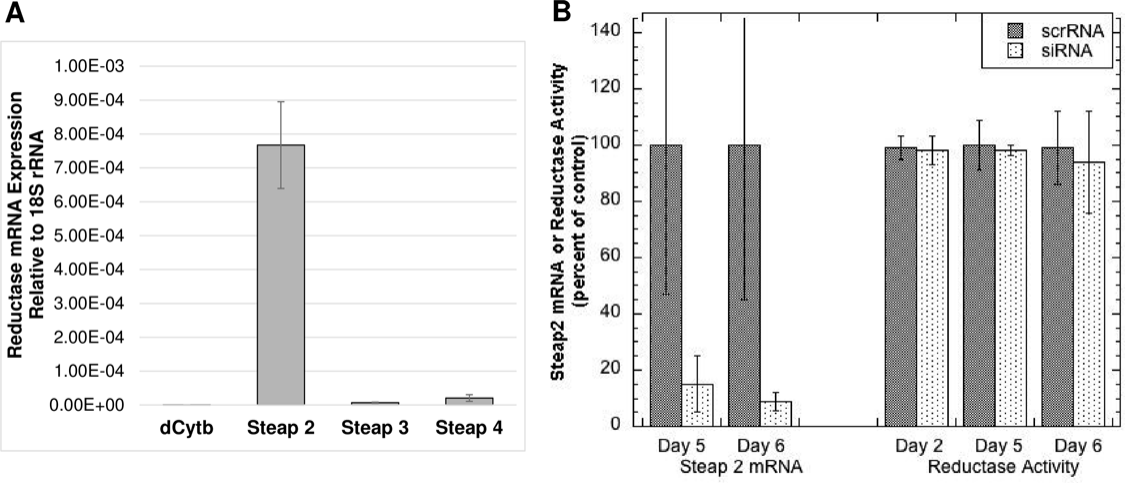
Expression of reductase mRNAs by MEF cells, and effect of Steap 2 siRNA knockdown on mRNA and copper reductase activity. (A) WT and Ctr1-null MEF cells were analyzed for expression of mRNA for dCytb, and Steaps 2, 3 and 4 by quantitative PCR, relative to 18S rRNA [expression = 2^(-∆Ct)]. (B) Effect of Steap 2 siRNA treatment (2 x over 5–6 days) on Steap 2 mRNA levels (relative to 18S RNA) compared to that of scrambled siRNA (ScrRNA), and on cell surface Cu(II) reductase activity, pooling results from two separate experiments, thus given as percent (N = 3 for each) of control (untreated) cells in the same experiment. Data are Means ± SD, N = 6. *p<0.001 for difference from control (ScrRNA).

To identify which reductase might be operating in these cells so that its expression could be knocked down, levels of mRNA for dCytB, and Steaps 2, 3 and 4 were measured by quantitative PCR. As shown in Fig 9A, only expression of Steap 2 was detected in the Ctr1+/+ and Ctr1-/- MEFs. Attempts were then made to knock down Steap 2, using siRNA. siRNA treatments reduced Steap2 mRNA levels markedly over 3–6 days (with treatments every 3 days) (Fig 9B). However, there was no substantial change in cell surface reductase activity, implying there was no drop in the level of Steap 2 protein. This indicates either that Steap2 has a long half-life or that it is not involved in mediating uptake of copper from Cp. So other approaches to knock down or knock out the reductase will have to be developed and applied, to determine whether or not this (or other) reductases are involved in Cp-Cu uptake.

## Discussion

As described in the Introduction, previous studies from our laboratory had provided evidence that ceruloplasmin (Cp), the main copper-binding protein in the blood plasma, is a circulating source of copper for many tissues in the mammalian organism, and particularly for heart, placenta, and the fetus (1,53,54), the main evidence being that radioactive copper in Cp (not exchangeable but buried in its structure) accumulated in most organs over time. This could have occurred through direct interaction of ceruloplasmin with cells in the various organs leading to docking with copper uptake transporters and/or endocytosis, and/or effects of other factors on the cell surface or in extracellular fluid, mediating release of Cu from Cp or otherwise mediating its cell uptake. The objectives of the studies presented here were to determine unequivocally that copper from Cp is or is not taken into cells, and if so to test the idea that this happens by direct interaction of purified Cp with transporters/receptors at the cell surface that leads to separation of the copper from holo Cp, the protein portion now becoming apo Cp and the copper entering the cell cytosol through CTR1 and/or the as yet unidentified copper transporter present in Ctr1 knockout fibroblasts [67,70]. The data reported here for cultured embryonic mouse fibroblasts and mammary epithelial cells provide conclusive evidence that the copper in Cp does enter the cell, and that this occurs through direct interaction of Cp with the cell surface resulting in copper uptake through copper transporters (including Ctr1), and formation of apo Cp.

Several different experimental approaches were used to demonstrate that the copper in Cp actually entered the cells with which it was incubated and did not just stick to the cell surface or cell surface receptors previously identified. Radioactive copper, which was buried in the structure of the purified Cp, accumulated in the cultured fibroblasts in linear fashion over time, and labeling was greatly reduced at 2° C. These are characteristic of an active uptake process (as opposed to reaching equilibrium of binding with the outside of a cell). In the case of the mammary epithelial cell monolayers grown on filters in bicameral chambers, radioactive copper from Cp applied to the basolateral (blood) side not only entered the monolayer but was increasingly released on the apical side over time, as part of milk secretions generated by the monolayer. Since the monolayer consists of polarized cells with tight junctions impermeable to diffusion of proteins, copper, and other constituents across the monolayer (down a concentration gradient between cells) [45], radioactive copper appearing in the milk secretions must have passed through the cells themselves. The radioactive copper from Cp associated with the fibroblasts could not be washed away–either by repeated washings with benign solutions or even with those of low pH, nor could it be removed by significant exposure of cells to trypsin–which would degrade proteins bound to and protruding from the cell plasma membrane. In washed cells broken open through bomb-cavitation or hypotonicity, more than 90% of the radioactive copper did not sediment with the membranous fraction but was in the cytosol, after ultracentrifugation. Although it is possible that some of the copper redistributed after cell lysis, most of the radioactive copper was in the interior of the cell in the cytosol.

These various findings indicated that in order for the Cu from Cp to enter cells, there had to be a direct interaction: The copper in Cp is not dialyzable or exchangeable, and yet it entered cells. So the interaction with cells made that possible. The culture medium was MEM and did not contain FBS–which might have provided other components to mediate uptake. The Cp itself was quite pure and was the only component labeled with radioactive copper. More importantly, we showed that during incubation with cells (but not in the absence of cells), there was a marked loss of Cu-containing holo Cp and an increase in the proportion of apo Cp. This indicates that the cells–and not something else in the culture medium or purified Cp, were responsible for the loss of holo Cp (removal of copper) and production of apo Cp. The total amount of Cp protein did not change, as determined by SDS-PAGE and immunoblotting. Moreover, the holo Cp was stable during the 24 h incubations in MEM; there was no decrease in holo Cp.

The finding that interaction of Cp with the cells caused the loss of holo Cp and generated apoCp–and without changing the amount of total Cp protein (as might occur if Cp were removed by the cells) allowed us to conclude as well that the uptake process involved separation of copper from Cp protein at the cell surface and not within the cell after uptake. The same conclusion was confirmed by other means, namely by showing that a broad range of endocytosis inhibitors failed to reduce rates of Cu uptake from Cp.

Thus indisputably, the Cu in Cp enters cells by direct interaction of Cp with the cell surface that results in release of Cp copper to uptake transporters, with formation of apo Cp; and Cp is legitimately to be considered not only as an enzyme and radical scavenger but also as a circulating copper transport protein bringing copper from the liver to cells in other organs. Our findings are consistent with and extend previous findings (reported by us and others) that led to the same overall conclusions, as already described in the Introduction. They are also compatible with earlier reports of specific binding of Cp to whole cells and plasma membrane preparations i.e. “Cp receptors” [15,56,58,60,63–65]. In those studies, binding was demonstrated with Cu-radiolabeled Cp protein at 2-4° C, and its specificity was validated by showing that a large excess of unlabeled Cp prevented a major portion of the binding. (There was also some non-specific binding.) Maximal binding/equilibration occurred within 1 h at 2-4° C [60]. Where studied, dissociation constants were in the range of 10^−8^ M (or 10 nM) [60,63,65]. This means that under normal physiological conditions, with a total Cp concentration in plasma of about 28 μM (half of which might be holo Cp [38,39,40]), the specific binding sites for Cp would be saturated. Such affinity and saturation would be expected if Cp were active in delivering copper to cells from the circulation. Binding would of course be the first step in the interaction of Cp with the cells to deliver copper to a copper transporter in the plasma membrane. However (and as might be expected), our preliminary data on the kinetics of uptake of Cu from Cp suggest that binding is not the rate-limiting step. Rates of uptake measured at Cp concentrations about 7-fold lower than physiological (in the range of 0.5–2 μM holoCp) were well below the rate of uptake Km. The latter is thus much higher than the dissociation constant for specific Cp receptor binding [60,63,65].

Consistent with receptor binding not being the rate-limiting step for uptake of Cu from Cp, it seems highly likely that a reductase is needed to provide Cu(I) for uptake. At any given moment (under physiological and cell culture conditions) the copper atoms in Cp would mostly be in the Cu(II) state, whereas CTR1 and the unknown uptake transporter in the MEFs take up Cu(I) [3,70]. Thus, reduction of the metal ion would have to intervene between binding of Cp and actual uptake of its copper into cells. In the case of enterocytes, reductases in the apical brush border are required for absorption/uptake of dietary copper (and iron) ions by the intestinal mucosa [74–76]. We had previously shown that copper and iron reductase activities are present on the cell surface of the fibroblasts used here [70]. If this activity is necessary for uptake of copper from Cp, uptake of radioactive Cu from Cp by the fibroblasts would be inhibited by an excess of non-radioactive Cu(II) (competing for reduction), and this is what we found. Similarly, if the reduced radioactive copper resulting from reduction of ^64^Cu in Cp now entered the cell through CTR1 or the unknown copper transporter, an excess of non-radioactive Cu(I) would compete for uptake, reducing the rate of entry of Cp-derived radioactive copper. Again our prediction was correct. In high concentrations, both forms of non-radioactive copper greatly lowered the rate of ^64^Cu uptake from Cp. Thus, it seems very likely that binding of Cp to the cell surface at least partly involves an interaction with a reductase, and that the transporter(s) receiving the resulting Cu(I) is/are close by. (Perhaps there is even a ternary complex of Cp, reductase, and transporter.) This is currently being explored. Since the known reductases also reduce Fe(III) [69,74], we predicted and found that an excess of Fe(III) markedly inhibited uptake of Cp-^64^Cu by the cells, providing further evidence that a reductase was mediating copper uptake from Cp. This is consistent as well with the results of other cellular copper uptake studies we have carried out, in which the ^64^Cu-labeled copper was delivered to the same kinds of MEF cells by albumin or transcuprein (alpha-2-macroglobulin) [70].

As to whether Cp has a special connection to CTR1, it appears this is not the case. Our data support the ability of CTR1 to take up Cu coming from Cp in that uptake by mammary epithelial cells was inhibited by Ag(I), and that in one stage of their evolution in our laboratory, MEFs expressing Ctr1 took up Cu significantly faster than those lacking Ctr1. However, Ctr1-null MEF cells also readily absorbed Cu from Cp, indicating that another as yet unidentified Cu uptake transporter could do the same thing [67,70]. This apparent lack of specificity may reflect the need for interaction of Cp not so much with a transporter but with a reductase and/or receptor, as we expect to determine in the future.

The striking loss of holo Cp during incubation with cells but not when cells were absent indicates that holo Cp is relatively stable in solution (when not interacting with cells), and confirms the well-established non-dialyzability of Cu in Cp. Our studies showing conversion of holo to apo Cp by the cells also indicate that this *in vivo* process is “all or none”. There are no obvious intermediates, at least as can be observed by immunoblotting of fresh blood plasma samples and/or of purified Cp after native PAGE, as has been shown by us and others [38–40]. [Whole plasma subjected to the established procedure for detecting apo and holo Cp–as used here, shows two clear-cut bands, the slower migrating one having no Cu, the faster one having Cu, and their migrations correspond to those of apo and holo Cp standards.] The results imply that the interaction of holo Cp with the cells leads to a major unraveling of structure that completely releases the copper ions from their various binding sites in the molecule [13,19], whereas *in vitro*, the ions in Type 1 (blue copper) sites appear to be more vulnerable to removal. In solution (without cells), holo Cp undergoes subtle changes in structure over time [15]. This involves formation of a proposed intermediate that has lost 1–2 copper atoms (but not from the trinuclear cluster) and retains enzymic activity [15,77], while conversion to apo Cp takes much longer. For example, using purified human Cp and CD, DSC as well as fluorescence analyses, Sedlak et al. [77] reported that the rate of holo Cp conversion of Cp to this intermediate took less than 24 h at 37° C and pH 7 (the composition of the solution was not specified). Further conversion (in the absence of cells) to apo Cp had a half-life of about 17 days. Apo Cp (produced by harsher treatment of holo Cp (namely with ascorbate and ferrocyanide) also underwent subtle changes, including formation of an intermediate, with an overall conversion rate half-life of about 2 days. As most of our studies were carried out with relatively fresh Cp incubated with cells in culture medium closer to physiological conditions over 3–6 h, it was most probably in a state comparable to that found *in vivo*, where only apo and holo Cp forms have been detected [38–40]. In the case where incubation was for 24 h to follow loss of holo Cp, some conversion to an intermediate (enzymatically active) form may have occurred, but this was not evident from its migration in native PAGE.
